# Supplementary material for: Reticulon proteins modulate autophagy of the endoplasmic reticulum in maize endosperm
Source: eLife. 2020 Feb 3;9:e51918. doi: 10.7554/eLife.51918 (PMC7046470; doi:10.7554/eLife.51918)
Supplement: Supplementary file 1. [file elife-51918-supp1.docx]

Supplementary File 1: Key Resources Table

| **Key Resources Table** | | | | |
| --- | --- | --- | --- | --- |
| **Reagent type (species) or resource** | **Designation** | **Source or reference** | **Identifiers** | **Additional information** |
| gene (Zea mays) | *Rtn1* |  | Zm00001d043551 |  |
| Gene (Zea mays) | *Rtn2* |  | Zm00001d012776 |  |
| Gene (Zea mays) | *Atg8a* |  | Zm00001d006474 |  |
| Genetic reagent (mutant) | *rtn1-1* | Maize Genetics Cooperation Stock Center | Mu-08559 | UniformMu line |
| Genetic reagent (mutant) | *rtn2-1* | Maize Genetics Cooperation Stock Center | Mu-02254 | UniformMu line |
| Genetic reagent (mutant) | *rnt2-2* | Maize Genetics Cooperation Stock Center | Mu-03723 | UniformMu line |
| Genetic reagent (mutant) | *rnt2-3* | Maize Genetics Cooperation Stock Center | Mu-00381 | UniformMu line |
| Genetic reagent (transgenic *Zea mays*) | *YFP-ATG8a* | (Lit et al 2015) |  |  |
| Transfected construct  (plants) | pRTL2-mCherry | Arabidopsis Biological Resource Center | CD3-1062 |  |
| Commercial assay, kit | Split-ubiquitin Y2H assay | Dualsystems, Biotech | P01501 |  |
| Commercial assay, kit | ProQuest Y2H system | Thermo Fisher Scientific | PQ10001-01 |  |
| Commercial assay, kit | High Capacity cDNA Reverse Transcription Kit | Applied Biosystems by Thermos Fisher Scientific |  |  |
| Recombinant DNA reagent | pDOE8-MAS:CFP-KDEL  (plasmid) | Gooking and Assman 2014 |  |  |
| Antibody | anti-Aca2  (Rabbit polyclonal) | Harper et al 1998 |  | WB (1:1000) |
| Antibody | Anti-Atg8  (Rabbit polyclonal) | Thompson et al 2005 |  | WB (1:500) |
| Antibody | anti-Bip  (Rabbit polyclonal) | Holding et al 2007 |  | WB (1:3000) |
| Antibody | anti-calnexin/calreticulin  (Rabbit polyclonal) | Pagny et al 2000 |  | WB (1:5000) |
| Antibody | anti-GFP  (Mouse monoclonal) | Sigma | 11814460001 | WB (1:5000) |
| Antibody | anti-HA  (Mouse monoclonal) | Sigma | H6908 | WB (1:5000) |
| Antibody | anti-H3  (Rabbit polyclonal) | AbCam | AB1791 | WB (1:3000 for protoplasts and 1:10000 for endosperm samples) |
| Antibody | anti-Nbr1  (Rabbit polyclonal) | McLoughlin et al., 2019 |  | WB (1:1000) |
| Antibody | Anti-Pdi  (Rabbit polyclonal) | (Li and Larkins, 1996 |  | WB (1:5000) |
| Antibody | anti-Rpt4  (Rabbit polyclonal) | Marshall et al., 2015 |  | WB (1:3000) |
| Antibody | anti-Vdac  (Rabbit polyclonal) | (Subbaiah et al., 2006 |  | WB (1:1000) |
| Antibody | goat anti-mouse HRP conjugate | SeraCare | 074-1806 | WB (1:5000-10000) |
| Antibody | goat anti-rabbit HRP conjugate | SeraCare | 074-1506 | WB (1:5000-10000) |
| Antibody | rabbit anti-chicken IgY HRP conjugate | Rancour et al., 2004 |  | WB (1:3000) |
| Software, algorithm | ImageJ software | Abràmoff et al., 2004 |  |  |
| Software, algorithm | TotalLab software | Non-linear Dynamics |  |  |
| sequence-based reagent | *Zm rtn1-1L* | This paper | PCR primer for plant genotyping | GAGGACCCCTTCTTCTCGTC |
| sequence-based reagent | *Zm rtn1-1R* | This paper | PCR primer for plant genotyping | CGTAGTTCGACGGTCAGGAT |
| sequence-based reagent | *Zm rtn2-1L* | This paper | PCR primer for plant genotyping | AGAATCGGGTATACAAATCGGGGG |
| sequence-based reagent | *Zm rtn2-1R* | This paper | PCR primer for plant genotyping | TGCCCAGCTGTTTTATAAGGGA |
| sequence-based reagent | *Zm rtn2-2L* | This paper | PCR primer for plant genotyping | TGCCCAGCTGTTTTATAAGGGA |
| sequence-based reagent | *Zm rtn2-2R* | This paper | PCR primer for plant genotyping | ATGTCCAATCTCCCTCAAGGTAGCAA |
| sequence-based reagent | *Zm rtn2-3L* | This paper | PCR primer for plant genotyping | AAGGTAGCAAAGCCCCTGTT |
| sequence-based reagent | *Zm rtn2-3R* | This paper | PCR primer for plant genotyping | AAGGTAGCAAAGCCCCTGTT |
| sequence-based reagent | *Zm rtn1-1L* | This paper | Primer for RT-PCR | CGTAGTTCGACGGTCAGGAT |
| sequence-based reagent | *Zm rtn1-1R* | This paper | Primer for RT-PCR | TCCAATCTCCCTCAAGGTAGCAAA |
| sequence-based reagent | *Zm rtn2-1L* | This paper | Primer for RT-PCR | CTCTCCTACACCTCCATTTCGTCGAA |
| sequence-based reagent | *Zm rtn2-1R* | This paper | Primer for RT-PCR | CTAGTGCTTCTTATCCTTGAGGCCC |
| sequence-based reagent | *Zm rtn2-2L* | This paper | Primer for RT-PCR | GTCCGCTCTTCGTCTATAATGGC |
| sequence-based reagent | *Zm rtn2-2R* | This paper | Primer for RT-PCR | CTAGTGCTTCTTATCCTTGAGGCCC |
| sequence-based reagent | *Zm rtn2-3L* | This paper | Primer for RT-PCR | TGCCCAGCTGTTTTATAAGGGAGAA |
| sequence-based reagent | *Zm rtn2-3R* | This paper | Primer for RT-PCR | TCCAATCTCCCTCAAGGTAGCAAA |
| sequence-based reagent | RTN2N80F | This paper | PCR primer for plasmid construction | ATGGCCGACCATAAGGAGGAGCA |
| sequence-based reagent | RTN2N80R | This paper | PCR primer for plasmid construction | CTAGATCTTCTTGTTCCTCCATAGGACAA |
| sequence-based reagent | RTN2L50F | This paper | PCR primer for plasmid construction | ATGTCCAGTTTCATCAACAAGTCCCCA |
| sequence-based reagent | RTN2L50R | This paper | PCR primer for plasmid construction | CTATTTCTTTAGATCGTGTCCATGTCCA |
| sequence-based reagent | RTN2C48F1 | This paper | PCR primer for plasmid construction | ATGACTATACCTGTTCTGTATGAG |
| sequence-based reagent | RTN2C48R1 | This paper | PCR primer for plasmid construction | CTAGTGCTTCTTATCCTTGAGGCCC |
| sequence-based reagent | RTN2C48F2 | This paper | PCR primer for plasmid construction | ATGACTATACCTGTTCTGTATGAGAAATATG |
| sequence-based reagent | RTN2N80Fr | This paper | PCR primer for plasmid construction | GGGGACAAGTTTGTACAAAAAAGCAGGCTCCATGGCCGACCATAAGGAGGAGCA |
| sequence-based reagent | RTN2N80Rr | This paper | PCR primer for plasmid construction | GGGGACCACTTTGTACAAGAAAGCTGGTCCTAGATCTTCTTGTTCCTCCATAGGACAA |
| sequence-based reagent | RTN2L50Fr | This paper | PCR primer for plasmid construction | GGGGACAAGTTTGTACAAAAAAGCAGGCTCCATGTCCAGTTTCATCAACAAGTCCCCA |
| sequence-based reagent | RTN2L50Rr | This paper | PCR primer for plasmid construction | GGGGACCACTTTGTACAAGAAAGCTGGTCCTATTTCTTTAGATCGTGTCCATGTCCA |
| sequence-based reagent | RTN2C48Fr | This paper | PCR primer for plasmid construction | GGGGACAAGTTTGTACAAAAAAGCAGGCTCCATGACTATACCTGTTCTGTATGAG |
| sequence-based reagent | RTN2C48Rr | This paper | PCR primer for plasmid construction | GGGGACCACTTTGTACAAGAAAGCTGGTCCTAGTGCTTCTTATCCTTGAGGCCC |
| sequence-based reagent | pBT3C-RTN2F | This paper | PCR primer for plasmid construction | ATTAACAAGGCCATTACGGCCAAAAATGGCCGACCATAAGGAGGAGCAG |
| sequence-based reagent | pBT3C-RTN2R | This paper | PCR primer for plasmid construction | AACTGATTGGCCGAGGCGGCCCCGTGCTTCTTATCCTTGAGGCCCT |
| sequence-based reagent | pPR3N-RTN2F | This paper | PCR primer for plasmid construction | ATTAACAAGGCCATTACGGCCATGGCCGACCATAAGGAGGAGCAG |
| sequence-based reagent | pPR3N-RTN2R | This paper | PCR primer for plasmid construction | AACTGATTGGCCGAGGCGGCCCTAGTGCTTCTTATCCTTGAGGCCCT |
| sequence-based reagent | pBT3C-RTN1F | This paper | PCR primer for plasmid construction | ATTAACAAGGCCATTACGGCCAAAAATGGCCGACCACAAGGAGGAGCCTG |
| sequence-based reagent | pBT3C-RTN1R | This paper | PCR primer for plasmid construction | AACTGATTGGCCGAGGCGGCCCCGTGCTTCTTATCCTTAAGGGGGCCCT |
| sequence-based reagent | pPR3N-RTN1F | This paper | PCR primer for plasmid construction | ATTAACAAGGCCATTACGGCCATGGCCGACCACAAGGAGGAGCCTG |
| sequence-based reagent | pPR3N-RTN1R | This paper | PCR primer for plasmid construction | AACTGATTGGCCGAGGCGGCCCTAGTGCTTCTTATCCTTAAGGGGGCCCT |
| sequence-based reagent | pPR3N-ATG8aF | This paper | PCR primer for plasmid construction | ATTAACAAGGCCATTACGGCCATGGCCAGGACCTCTTTCAAAATGG |
| sequence-based reagent | pPR3N-ATG8aR | This paper | PCR primer for plasmid construction | AACTGATTGGCCGAGGCGGCCC TAGGCAGAGCCGAAGGTGTTTTCACC |
| sequence-based reagent | HA-RTN2cDNA F | This paper | PCR primer for plasmid construction | CGAAGCTTATGTACCCATACGATGTTCCAGATTACGCTATGGCCGACCATAAGGAGGAGCAGTC |
| sequence-based reagent | Bam H I-RTN2cDNA R | This paper | PCR primer for plasmid construction | TAGGATCC CTAGTGCTTCTTATCCTTGAGGCC |
| sequence-based reagent | RTN2-HAcDNA F | This paper | PCR primer for plasmid construction | CGAAGCTT ATGGCCGACCATAAGGAGGAGCAGTC |
| sequence-based reagent | RTN2HAcDNA R | This paper | PCR primer for plasmid construction | TAGGATCC CTA AGCGTAATCTGGAACATCGTATGGGTA GTGCTTCTTATCCTTGAGGCC |
| sequence-based reagent | HA-RTN1cDNA F | This paper | PCR primer for plasmid construction | CGAAGCTTATGTAC CCATACGATGTTCCAGATTACGCTATGGCCGACCACAAGGAGGAGCCTGTG |
| sequence-based reagent | Bam H I-RTN1cDNA R | This paper | PCR primer for plasmid construction | TAGGATCCCTAGTGCTTCTTATCCTTAAGG |
| sequence-based reagent | RTN1HAcDNA F | This paper | PCR primer for plasmid construction | CGAAGCTT ATGGCCGACCACAAGGAGGAGCCTGTG |
| sequence-based reagent | RTN1HAcDNA R | This paper | PCR primer for plasmid construction | TAGGATCC CTA AGCGTAATCTGGAACATCGTATGGGTA GTGCTTCTTATCCTTAAGG |
| sequence-based reagent | RTN2-GFP R | This paper | PCR primer for plasmid construction | GTGAAAAGTTCTCCTTTACCCATGTGCTTCTTATCCTTGAGGCCC |
| sequence-based reagent | RTN2-GFP F | This paper | PCR primer for plasmid construction | GGGCCTCAAGGATAAGAAGCAC ATGGGTAAAGGAGAACTTTTCAC |
| sequence-based reagent | BamH I-GFP R | This paper | PCR primer for plasmid construction | TAGGATCC TTAAGATCTGTATAGTTCGTCCATGCC |
| sequence-based reagent | RTN1-GFP R | This paper | PCR primer for plasmid construction | GTGAAAAGTTCTCCTTTACCCATGTGCTTCTTATCCTTAAGGGGGCCC |
| sequence-based reagent | RTN1-GFP F | This paper | PCR primer for plasmid construction | GGGCCCCCTTAAGGATAAGAAGCAC ATGGGTAAAGGAGAACTTTTCAC |
| sequence-based reagent | pPR3N-RTN2 MuR | This paper | PCR primer for plasmid construction | TCGAAAGCGGCCGCTGCAGCCTTCAGTTCGATCATAGCCTTCTC |
| sequence-based reagent | PR3N-RTN2 MuF | This paper | PCR primer for plasmid construction | TGAAGGCTGCAGCGGCCGCTTTCGATGAGAAGTGCCTATCG |
| sequence-based reagent | pPR3N-RTN1 MuR | This paper | PCR primer for plasmid construction | TCGAA AGCGGCCGCTGCAGCCTTCAGTTCAGTCATAGCCTTCTC |
| sequence-based reagent | PR3N-RTN1 MuF | This paper | PCR primer for plasmid construction | TGAAG GCTGCAGCGGCCGCT TTCGA TGAGAAGTGCCTATCGAAG |
| sequence-based reagent | RTN2 12A F | This paper | PCR primer for plasmid construction | GCGGCAGCTGCAGCCGCGAGGGAGATTGGACATGGACACGATCTAGCGGCAGCCGCAGCTGCT |
| sequence-based reagent | RTN2 12A R | This paper | PCR primer for plasmid construction | AGCAGCTGCGGCTGCCGCTAGATCGTGTCCATGTCCAATCTCCCTCGCGGCTGCAGCTGCCGC |
| sequence-based reagent | RTN2 6ALoop F | This paper | PCR primer for plasmid construction | GCG GCA GCT GCA GCC GCG AGGGAGATTGGACATGGACACG |
| sequence-based reagent | RTN2 6ALoop R | This paper | PCR primer for plasmid construction | CGTGTCCATGTCCAATCTCCCTCGCGGCTGCAGCTGCCGC |
| sequence-based reagent | RTN2 6ATM F | This paper | PCR primer for plasmid construction | GGGAGATTGGACATGGACACGATCTAGCGGCAGCCGCAGCTGCT |
| sequence-based reagent | RTN2 6ATM R | This paper | PCR primer for plasmid construction | AGCAGCTGCGGCTGCCGCTAGATCGTGTCCATGTCCAATCTCCC |
| sequence-based reagent | RTN2 12A R2 | This paper | PCR primer for plasmid construction | CGCGGCTGCAGCTGCCGCGTTGATCTCGTATCTCAGTGTCAG |
| sequence-based reagent | RTN2 12A F2 | This paper | PCR primer for plasmid construction | GCGGCAGCCGCAGCTGCTATCGCAGGTCTCTGGCTCTTTTCAG |
| sequence-based reagent | mCherry F | This paper | PCR primer for plasmid construction | GGGCCATGATAAGCAAGGGCGAGGAGGATAACATGG |
| sequence-based reagent | Xba I-mCherry R | This paper | PCR primer for plasmid construction | TATCTAGATCACAGCTCGTCATGCAGATCTAGTCCGGACTTGTAC |
| sequence-based reagent | RTN2mCherryR2 | This paper | PCR primer for plasmid construction | TTATCCTCCTCGCCCTTGCTTATCATGTGCTTCTTATCCTTGAGGCCCTTTGG |
| sequence-based reagent | RTN2mCherryF3 | This paper | PCR primer for plasmid construction | CCAAAGGGCCTCAAGGATAAGAAGCACATGATAAGCAAGGGCGAGGAGGATAA |
| sequence-based reagent | RTN2mCherryR4 | This paper | PCR primer for plasmid construction | TATCTAGATCACAGATCTAGTCCGGACTTGTACAG |
| sequence-based reagent | RTN2 Mu4C R2 | This paper | PCR primer for plasmid construction | CAGCGAGACGATGAGAGCGTGGGCCACCAAGGTGAGGAGATGGTACTCC |
| sequence-based reagent | RTN2 Mu4C F3 | This paper | PCR primer for plasmid construction | GCCCACGCTCTCATCGTCTCGCTG GCCATCCTGTTCC |
| sequence-based reagent | RTN2 Mu4C R4 | This paper | PCR primer for plasmid construction | GAACAGTGTCAGGAAGTTCGCTGCGCTCCCAAGAACTGAAAAGAGCC |
| sequence-based reagent | RTN2 Mu4C F5 | This paper | PCR primer for plasmid construction | GCAGCGAACTTCCTGACACTGTTCTATATTGTC |
| sequence-based reagent | 35S-HA-RTN2cDNA F | This paper | PCR primer for plasmid construction | GGGCCATGTACCCATACGATGTTCCAGATTACGCT |
| sequence-based reagent | 35S-RTN2cDNA R | This paper | PCR primer for plasmid construction | TATCTAGACTAGTGCTTCTTATCCTTGAGGCC |
| sequence-based reagent | RTN2HAcDNA R | This paper | PCR primer for plasmid construction | TATCTAGACTAAGCGTAATCTGGAACATCGTATGGGTA |
| sequence-based reagent | SG-mCherry-HDEL F | This paper | PCR primer for plasmid construction | GGGCCATGAAGGTACAGGAGGGT |
| sequence-based reagent | Atg8aMuYL F | This paper | PCR primer for plasmid construction | GATAAGAAGAAGGCCGCTGTCCCTGCTGATCTCACTGTCGG |
| sequence-based reagent | Atg8aMuYL R | This paper | PCR primer for plasmid construction | GATCAGCAGGGACAGCGGCCTTCTTCTTATCAATCTCGGGGAC |
| sequence-based reagent | Atg8a MuG R | This paper | PCR primer for plasmid construction | AACTGATTGGCCGAGGCGGCCCTAGGCAGAGGCGAAGGTGTTTTCACC |
| sequence-based reagent | RTN2TM12 R2 | This paper | PCR primer for plasmid construction | AGCAGCTGCCGCTGCCGCTAGATCGTGTCCATGTCCAATCTCCC |
| sequence-based reagent | RTN2TM12 F3 | This paper | PCR primer for plasmid construction | GCGGCAGCGGCAGCTGCT ATCGCAGGTCTCTGGCTC |
| sequence-based reagent | RTN2TM12 R4 | This paper | PCR primer for plasmid construction | CGCTGCCGCAGCGGCAGC ATAGAACAGTGT CAGGAAGTTGC RTM2TM12 F5 |
| sequence-based reagent | RTM2TM12 F5 | This paper | PCR primer for plasmid construction | GCTGCCGCTGCGGCAGCGTACACTATACCTGTTCTGTATGAG |
| sequence-based reagent | RTN2Qua R7 | This paper | PCR primer for plasmid construction | AGCGGCTGCCGCCGCAGCAATGTTTGGTGGGGACTTG |
| sequence-based reagent | RTN2Qua F8 | This paper | PCR primer for plasmid construction | GCTGCGGCGGCAGCCGCT GAGGATGTGTCTGTTAATGTTGC |
| sequence-based reagent | RTN2Qua R7b | This paper | PCR primer for plasmid construction | CCTCGCGGCAGCAGCGGCCGCGTTGATCTCGTATCTCAGTGTCAG |
| sequence-based reagent | RTN2Qua F8b | This paper | PCR primer for plasmid construction | GCGGCCGCTGCTGCCGCGAGGGAGATTGGACATGGACACG |
| sequence-based reagent | RTN1eGFP F1 | This paper | PCR primer for plasmid construction | GGGCCATGGCCGACCACAAGGAGGAGCCTG |
| sequence-based reagent | RTN1eGFP R1 | This paper | PCR primer for plasmid construction | CCAGTGAAAAGTTCTCCTTTACCCAT GTGCTTCTTATCCTTAAGGGGGCCC |
| sequence-based reagent | RTN1eGFP F2 | This paper | PCR primer for plasmid construction | GGGCCCCCTTAAGGATAAGAAGCAC ATGGGTAAAGGAGAACTTTTCACTGG |
| sequence-based reagent | RTN1_3'_F1 | This paper | Primer for qRT-PCR | ATCTGTATGGGCAACTTTGGTGTG |
| sequence-based reagent | RTN1_3'_R1 | This paper | Primer for qRT-PCR | GCTTCCAGGCAACTATGGTGAA |
| sequence-based reagent | RTN2_3'_F1 | This paper | Primer for qRT-PCR | TCATGTGAACTTTGCCATACACCTT |
| sequence-based reagent | RTN2_3'_R1 | This paper | Primer for qRT-PCR | ACTAGACCAATTACTATCCCTGCGT |
| sequence-based reagent | ZmBIP2-F | This paper | Primer for qRT-PCR | CCAATGACCAGGGTAACCGTATC |
| sequence-based reagent | ZmBIP2-R | This paper | Primer for qRT-PCR | CTGAAGACCTTGTTCTCCCCATC |
| sequence-based reagent | EF1α-F | This paper | Primer for qRT-PCR | TGGGCCTACTGGTCTTACTACTGA |
| sequence-based reagent | EF1α-R | This paper | Primer for qRT-PCR | ACATACCCACGCTTCAGATCCT |
